# Draft Genome Sequences of Multidrug-Resistant Shiga Toxin-Producing Escherichia coli O116:H25 Strains from Ready-to-Eat Foods Sold in Lagos, Nigeria

**DOI:** 10.1128/mra.00314-22

**Published:** 2022-07-13

**Authors:** Oluwadamilola M. Makinde, Deidré Van Wyk, Odion O. Ikhimiukor, Carlos Bezuidenhout, Rasheed A. Adeleke, Chibundu N. Ezekiel

**Affiliations:** a Department of Microbiology, Babcock University, Ilishan Remo, Ogun State, Nigeria; b Unit for Environmental Sciences and Management, North-West University, Potchefstroom, South Africa; c Department of Biological Sciences, Southwestern University, Okun-Owa, Ogun State, Nigeria; d Environmental Microbiology and Biotechnology Laboratory, Department of Microbiology, University of Ibadan, Ibadan, Oyo State, Nigeria; University of Maryland School of Medicine

## Abstract

Draft genomes of multidrug-resistant Shiga toxin-producing Escherichia coli (STEC) strains IPK9(1) and IKS1(2), which were isolated from ready-to-eat foods (kokoro and shawarma) sold in Lagos, Nigeria, are reported. The genomes possessed genetic determinants for virulence and the antibiotic resistance gene for macrolide-associated resistance *mdf(A)*. Ready-to-eat foods increase public health threats in Nigeria.

## ANNOUNCEMENT

Bacteria are primary contaminants of ready-to-eat (RTE) foods (1), and strains resistant to antibiotics constitute a major public health concern. Here, we report the draft genomes of Shiga toxin-producing Escherichia coli (STEC) strains IPK9(1) and IKS1(2), which were isolated from kokoro and shawarma, respectively, sold in Lagos, Nigeria, and were screened phenotypically against eight antibiotics.

Five grams of RTE food samples were homogenized in 50 mL of peptone water for 2 min and centrifuged at 3,466 × *g* for 1 h. A 10-fold serial dilution was carried out with each homogenized sample. One milliliter of the homogenate was spread plated on nutrient agar (NA) (Sigma-Aldrich, Germany) and incubated at 37°C for 18 to 24 h. Pure colonies were subcultured on NA and subsequently on Mueller-Hinton agar for phenotypic antibiotic susceptibility testing using a set of eight antibiotics. The two strains recorded multiple antibiotic resistance index values (1) of 0.8 and 0.5, as described by CLSI guidelines (1), were subcultured in nutrient broth, and were grown overnight at 37°C in a shaking incubator at 220 rpm. The broth culture was centrifuged and washed in phosphate-buffered saline at 10,000 × *g*. Genomic DNA was extracted using the Quick-DNA fungal/bacterial miniprep kit (catalog number D6005; Zymo Research), following the manufacturer's protocol. The concentration and quality of DNA were determined using a Qubit 2.0 fluorometer (catalog number Q32866; Thermo Fisher Scientific, USA). Sequencing libraries were generated using a DNA preparation kit (Illumina, San Diego, CA) and sequenced on an Illumina MiSeq 2000 instrument with a v3 flow cell.

Raw sequence reads were quality filtered using Trimmomatic v0.36 (2). Adapter sequences were clipped using a mismatch value of 2, a palindrome clip threshold of 30, and a simple clip threshold of 10. The genomes were assembled using SPAdes v3.13.05 (3). Read quality and species designation were performed with Qualifyr v1.4.4 (https://gitlab.com/cgps/qualifyr) and Bactinspector v0.1.3 (https://gitlab.com/antunderwood/bactinspector). Genomes in FASTA file format were annotated using the NCBI Prokaryotic Genome Annotation Pipeline (PGAP) v5.3 (4). The basic features of each genome assembly are shown in Table 1.

**TABLE 1:** Basic characteristics of genome assemblies of the two multidrug-resistant STEC strains

Bacterial strain	No. of reads	Assembly size (bp)	No. of contigs	G+C content (%)	*N*_50_ (bp)	*L*_50_	Total no. of CDSs^*a*^	No. of protein CDSs	No. of pseudogenes	Total no. of RNAs
Escherichia coli IPK9(1)	369,118	4,700,727	66	50.7	196,516	8	4,492	4,369	123	96
Escherichia coli IKS1(2)	152,944	4,697,413	69	50.7	193,682	9	4,493	4,361	132	95

a CDS, coding sequence.

Default parameters for the publicly available Center for Genomic Epidemiology (www.cge.cbs.dtu.dk) pipelines ResFinder v4.1 (5), VirulenceFinder v2.0 (6), and SerotypeFinder v2.0 (7) were used to identify antimicrobial resistance genes, virulence genes, and serotype, respectively.

The strains possessed the virulence genes *lpfA* and *terC*, with *gad* being detected only in strain IKS1(2). The *mdf(A)* gene was the only acquired antibiotic resistance determinant, and it is responsible for resistance to numerous antibiotic classes (8–11). Both strains belong to STEC serotype O116:H25, are determined bioinformatically, and are diarrheagenic. These strains have been reported in surface water in British Columbia, Canada (12, 13), but not in RTE foods in Nigeria.

Poor food handling and personal hygiene practices during processing and selling of RTE foods to consumers have been associated with RTE contamination by bacteria in Nigeria (14). Multidrug-resistant STEC O116:H25 strains harboring virulence and antibiotic resistance [*mdf(A)*] determinants in RTE foods emphasize the potential hazards posed to consumers of RTE foods in Nigeria.

### Data availability.

The draft genome sequences reported in this study have been deposited in NCBI/GenBank under the accession numbers JAJCFG000000000 and JAJCFH000000000 for STEC strains IPK9(1) and IKS1(2), respectively. The SRA accession numbers for raw reads are SRX14686995 and SRX14686996 for IPK9(1) and IKS1(2), respectively.
